# Poisoning Deaths Involving Opioid Analgesics — New York State, 2003–2012

**Published:** 2015-04-17

**Authors:** Mark J. Sharp, Thomas A. Melnik

**Affiliations:** 1 Division of Information and Statistics, Office of Quality and Patient Safety, New York State Department of Health

Deaths involving opioid analgesics have increased dramatically in the United States. Approximately 4,000 such deaths were documented in 1999 (1), increasing to 16,235 in 2013, reflecting a nearly quadrupled death rate from 1.4 to 5.1 deaths per 100,000 (2). To investigate this increase in New York state, trends in poisoning deaths involving opioid analgesics from 2003 to 2012 were examined. Data sources used were New York state vital statistics multiple-cause-of-death data, consisting of data from both the New York City (NYC)* and non-NYC reporting jurisdictions, as well as statewide Medicaid enrollment data. Deaths involving opioid analgesics increased both in number and as a percentage of all drug poisoning deaths, and rates were highest among men, whites, persons aged 45–64 years, persons residing outside of NYC, and Medicaid enrollees. The analysis found that, in 2012, 70.7% of deaths involving opioid analgesics also involved at least one other drug, most frequently a benzodiazepine. These results underscore the potential to mitigate the trend of increasing opioid analgesic-related mortality through initiatives such as New York state’s Internet System for Tracking Over-Prescribing (I-STOP) law,† which took effect on August 27, 2013. Provisions under I-STOP include the requirements that providers consult the Prescription Monitoring Program (PMP) Registry when writing prescriptions for controlled substances, and that they use electronic prescribing.

New York state vital statistics multiple-cause-of-death mortality data, and statewide Medicaid enrollment data were used for this investigation. All reported rates were calculated using U.S. Census Bureau bridged-race population estimates (3) for New York state for each year included in this report. Age-adjusted rates were calculated using the direct method based on the 2000 U.S. standard population (4). Medicaid enrollment figures were used to compare rates between Medicaid recipients and non-recipients. Decedents were classified as Medicaid recipients if there was any record of Medicaid enrollment in the previous 12 months. Adopting a previously reported coding methodology (1), the *International Classification of Diseases, Tenth Revision* (ICD-10), codes used for identifying deaths with drug poisoning of any intent as an underlying cause were X40–44, X60–X64, X85, and Y10–Y14. Among these deaths, those involving opioid analgesics were identified using codes T40.2–T40.4, benzodiazepines using code T42.4, cocaine using T40.5, and heroin using T40.1. ICD-10 codes T36–T50.8 were used to identify specified drugs, with T50.9 being unspecified. Only deaths of New York state residents were included in the analysis.

From 2003 to 2012, the number of deaths with drug poisoning as an underlying cause increased from 750 to 1,869. During the same period, deaths involving opioid analgesics increased from 179 in 2003 to 883 in 2012 (Table). In addition, drug poisoning deaths without a drug specified, for which opioid analgesics might account partially, increased from 326 deaths in 2003 to 423 in 2012. Over this period, the percentage of drug deaths that involved opioid analgesics increased from 23.9% in 2003 to 47.2% in 2012, reaching a high of 54.0% in 2010.

Demographic differences were found in mortality involving opioid analgesics. Rates were consistently highest among New York state residents who were men, whites, non-NYC residents, and Medicaid enrollees (Table). Analysis of trends in crude death rates for poisonings involving opioid analgesics by age group showed rates were consistently highest among those aged 45–64 years, followed by those aged 20–44 (Figure 1). Rate ratios (RRs) comparing death rates between 2003 and 2012 (Table) indicate that the highest rate of increase in deaths involving opioid analgesics was among those aged 65–84 years (RR = 6.9). Persons in the race category “Other” and those residing in NYC also showed higher rates of increase in opioid analgesic-related mortality (RR = 6.3 and 7.5, respectively).

New York state Medicaid enrollees had higher death rates for opioid analgesic poisonings than did those not enrolled in Medicaid, and the differences increased over time (Table). Deaths per 100,000 among all New York state residents not enrolled in Medicaid increased from 0.73 in 2003 to 2.82 in 2012, while among Medicaid enrollees, the rates increased from 1.57 to 8.31 over the same period. Medicaid enrollees tend to be younger than persons not enrolled, and are more likely to be women. To control for the demographic differences between the two populations, age-adjusted death rates for poisonings involving opioid analgesics were stratified by sex (Figure 2). In each year and across both sexes, the age-adjusted death rates among Medicaid enrollees were higher than among those not enrolled in Medicaid.

Deaths involving opioid analgesics tend to involve multiple drugs. In 2012, of the 883 fatalities in New York state that involved opioid analgesics, 624 (70.7%) had at least one other specified drug documented on the death certificate, 27 (3.1%) involved an unspecified drug, with the remaining 232 (26.3%) opioid analgesic deaths involving no other drug. Benzodiazepines were the single most frequently documented drugs on the death certificate in addition to opioid analgesics. Of the 624 deaths involving opioid analgesics with at least one other specified drug, 308 (49.4%) involved benzodiazepines, followed by 153 (24.5%) involving cocaine, and 119 (19.1%) involving an unspecified antidepressant.

## Discussion

Increasing mortality associated with opioid analgesics has followed a similar upward trend in New York state and the nation since the late 1990s (2). While poisoning deaths involving any drugs have increased, opioid analgesics have accounted for an increasing proportion, with the percentage doubling over the 10-year period. Unlike the national trend of a more rapid increase in opioid analgesic-related deaths among women (5), the rate of increase, as indicated by the rate ratios, is slightly higher in New York state for men.

Comparison of opioid analgesic-related mortality between those enrolled or not enrolled in Medicaid shows considerably higher death rates and a more rapid increase in mortality among Medicaid enrollees. The consistently higher age-adjusted death rates for poisonings involving opioid analgesics among Medicaid enrollees (after stratifying data by sex) suggest that differences in age and sex distributions do not underlie these Medicaid/non-Medicaid differences. Other factors, such as the greater prevalence of mental illness and substance abuse in the Medicaid population (6), might contribute to the observed differences.

Deaths involving opioid analgesics in New York state tended to involve at least one other drug. In 2012, of the 883 drug poisoning fatalities in New York state that involved opioid analgesics, 624 (70.7%) had at least one other specified drug documented on the death certificate as having contributed to the death, and of these, 308 (49.4%) involved benzodiazepines. The tendency in New York state for opioid analgesic-related deaths to involve at least one other drug is greater than a national estimate of deaths in 2006, in which 51% of opioid analgesic-related deaths involved at least one other specified drug, with benzodiazepines involved in 17% of those deaths (7). It has been suggested that increases in opioid analgesic-related mortality might be related to an overall increase in prescribing these medications out of concern for the under-treatment of pain (8) accompanied by inappropriate prescribing and monitoring of patients to whom opioid analgesics are prescribed (9).

The findings in this report are subject to at least four limitations. First, heightened attention to the issue of opioid analgesic poisoning might have resulted in changes to reporting practices over time, increasing the likelihood of opioid involvement being reported on the death certificate. Second, geographic variation in cause of death determination or reporting could have influenced findings of regional differences in opioid analgesic-related mortality. Third, deaths involving opioid analgesics might have been undercounted because post-mortem drug test results might not have been available at the time the death certificate was completed. Finally, deaths associated with unspecified drugs account for a significant proportion of total drug mortality, resulting in the possibility that statistics for specific drug types, including opioid analgesics, are underestimated.

The increasing rates of opioid analgesic-related deaths among all groups, coupled with the multiple drug involvement in a high proportion of these deaths, suggest the need for a statewide system to prevent the abuse of prescription medications by ensuring that prescribers review a patient’s prescription history before prescribing these drugs. The recently implemented I-STOP initiative is an example. Such efforts to address the problem of opioid analgesic-related mortality might be especially important for the Medicaid population, in which prescription opioid-related deaths are more common.

What is already known on this topic?

Prescription drug abuse is an urgent public health problem facing the United States. Nationally, deaths caused by drug poisonings have increased over the last decade, with deaths associated with opioid analgesics showing the most rapid increases.

What is added by this report?

In New York state during 2003–2012, poisoning deaths involving opioid analgesics increased both in number and as a percentage of all drug poisoning deaths. Rates were highest among men, whites, persons aged 45–64 years, non-New York City residents, and Medicaid enrollees. In 2012, 70.7% of deaths involving an opioid analgesic also involved at least one other drug, most frequently a benzodiazepine.

What are the implications for public health practice?

Increasing mortality involving opioid analgesics and the multiple drug involvement in many of these deaths highlight the importance of efforts to ensure that prescribers of controlled substances consult a prescription registry for their patients’ histories of dispensed prescriptions for these medications. The New York state I-STOP law, with the requirements that prescribers consult the PMP Registry when writing prescriptions for controlled substances and that they use electronic prescribing, is one such effort. Such steps are especially important for Medicaid patients, who are at higher risk of opioid-associated poisoning death.

## Figures and Tables

**FIGURE 1:**
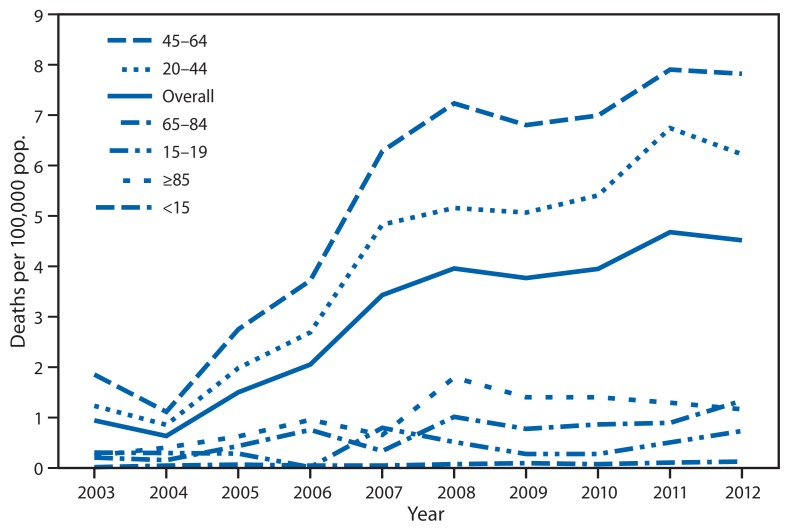
Death rates for poisonings involving opioid analgesics, by age group (yrs) — New York state, 2003–2012

**FIGURE 2:**
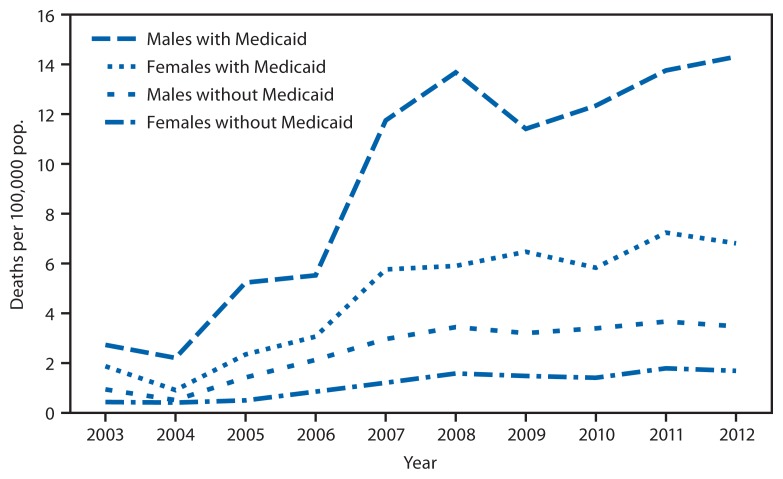
Age-adjusted death rates for poisonings involving opioid analgesics, by Medicaid enrollment status and sex — New York state, 2003–2012

**TABLE:** Number and crude death rates for poisonings involving opioid analgesics, by year and demographic characteristics — New York state, 2003–2012

Characteristic	Year										Ratio
	
	2003	2004	2005	2006	2007	2008	2009	2010	2011	2012	2012:2003
**Number of deaths**											
**All drug poisonings**	**750**	**522**	**891**	**952**	**1,581**	**1,679**	**1,566**	**1,415**	**1,853**	**1,869**	**2.5**
Drug poisonings involving opioid analgesics	179	120	287	394	660	769	734	764	909	883	4.9
**Opioid analgesics–related deaths per 100,000 population**											
**Total**	**0.93**	**0.62**	**1.49**	**2.04**	**3.42**	**3.95**	**3.76**	**3.94**	**4.67**	**4.51**	**4.8**
**Age group (yrs)**											
<15	0.00	0.03	0.05	0.03	0.03	0.06	0.08	0.06	0.09	0.11	-
15–19	0.23	0.39	0.61	0.94	0.64	1.78	1.39	1.39	1.28	1.15	5.0
20–44	1.22	0.84	1.97	2.68	4.82	5.15	5.06	5.40	6.74	6.22	5.1
45–64	1.84	1.10	2.74	3.71	6.28	7.23	6.8	6.99	7.90	7.82	4.3
65–84	0.19	0.14	0.42	0.74	0.32	1.00	0.76	0.85	0.88	1.32	6.9
≥85	0.29	0.28	0.27	0	0.78	0.50	0.26	0.26	0.49	0.72	2.5
**Sex**											
Females	0.70	0.48	0.88	1.35	2.26	2.57	2.69	2.58	3.39	3.18	4.5
Males	1.19	0.77	2.14	2.78	4.65	5.40	4.88	5.40	6.03	5.76	4.8
**Race**											
Black	0.45	0.20	0.61	0.93	1.85	1.78	2.10	2.21	2.36	2.48	5.5
White	1.12	0.76	1.81	2.47	4.07	4.79	4.45	4.77	5.67	5.39	4.8
Other	0.28	0.28	0.48	0.53	0.92	0.96	1.14	0.79	1.43	1.77	6.3
**Region**											
NYC	0.52	0.37	0.69	0.75	2.80	3.52	3.28	3.67	3.64	3.89	7.5
New York state, excluding NYC	1.23	0.81	2.08	2.99	3.88	4.27	4.13	4.14	5.43	4.98	4.0
**Medicaid status**											
Medicaid	1.57	1.06	2.74	3.22	6.50	7.23	6.81	7.06	8.40	8.31	5.3
Non-Medicaid	0.73	0.47	1.03	1.61	2.32	2.78	2.61	2.66	3.06	2.82	3.9

**Abbreviation:** NYC = New York City

## Footnotes

* New York City vital statistics data were provided by the New York City Department of Health and Mental Hygiene and used under the terms of a memorandum of understanding with the New York State Department of Health.

† Additional information at https://www.health.ny.gov/professionals/narcotic/prescription_monitoring/.
